# Understanding the association between immune-modulating helminths and HPV or cervical cancer: A scoping review

**DOI:** 10.1371/journal.pntd.0013308

**Published:** 2026-09-02

**Authors:** Allison Frank, Margarita Correa-Mendez, Alicia A. Livinski, Kalina Duncan, Eva H. Clark, Patti E. Gravitt

**Affiliations:** 1 Boston Medical Center, Boston, Massachusetts, United States of America; 2 Center for Global Health, National Cancer Institute, National Institutes of Health, Rockville, Maryland, United States of America; 3 National Institutes of Health Library, Office of Research Services, Office of the Director, National Institutes of Health, Bethesda, Maryland, United States of America; 4 Department of Medicine, Section of Infectious Diseases, Baylor College of Medicine, Houston, Texas, United States of America; 5 Georgia Cancer Center, Augusta University, Augusta, GeorgiaUnited States of America; Cyprus International University: Uluslararasi Kibris Universitesi, CYPRUS

## Abstract

**Background:**

Cervical cancer disproportionately affects people living in low- and middle-income countries (LMICs). While access to effective preventive interventions explains some of the disparity, biological causes cannot be ruled out as important contributors to the observed disparities in cervical cancer outcomes. Since chronic infection with helminths which cause immune dysregulation and suppress anti-viral responses is common in LMICs, we sought to review the available epidemiologic evidence evaluating associations between helminth infection and HPV infection and/or cervical cancer.

**Methods:**

We searched five databases of scientific publications using search terms targeted towards journal articles published between 1990–2022 evaluating an epidemiologic association between helminth infection and HPV prevalence, persistence, or cervical cancer progression. Out of 93 reports assessed for eligibility, we identified eight relevant studies and describe them in this scoping review.

**Results:**

All eight studies suggest a possible positive association between helminth infection and HPV or cervical cancer. Six studies found a positive trend in association between schistosomiasis and cervical neoplasia; two studies between hookworm infection and HPV prevalence; and 1 study between *Ascaris*, *Trichuris*, and *Strongyloides* and HPV prevalence.

**Conclusions:**

Published evidence suggests positive trends towards an association between helminth infection and cervical neoplasia. Given the high burden of helminth and HPV co-infection in LMICs, further evaluation of the association between helminth infection and HPV or cervical cancer is warranted.

## Author summary

The high prevalence of helminth and HPV co-infections in LMICs significantly impacts quality of life, necessitating studies to expand our knowledge of their epidemiological and health impacts. Understanding the interaction between helminth infection, persistence of high-risk HPV, and cervical cancer is then crucial for developing evidence-based public health programs to improve health outcomes in affected populations. Chronic helminth infections, such as schistosomiasis and hookworm, induce immune responses that can impair antiviral responses critical for HPV clearance. Eight studies included in this scoping review suggest a positive association between helminth infection and cervical pre-cancerous and cancerous lesions, warranting further evaluation of this interaction given the high burden of co-infection. Findings expand the evidence-base to support the need to incorporate schistosomiasis screening into gynecological examinations and/or cervical cancer screening efforts to enhance the accuracy of diagnosing and treating pre- and cancerous cervical lesions in endemic regions.

## Introduction

In 2020, globally, over 604,000 people were diagnosed with cervical cancer and over 341,000 deaths were attributed to the disease [1]. Although cervical cancer afflicts people across the globe, more than 85% of deaths occur in low- and middle-income countries (LMICs) [2]. Persistent infection with oncogenic human papillomavirus (HPV) types has been directly linked to cervical cancer development [3]. While high-risk HPV (hrHPV) infections become undetectable within 24 months in around 90% of infected individuals, hrHPV infections which remain persistently detectable have been shown to significantly increase the risk of progression to cancer [4–7]. Well-established risk factors associated with hrHPV persistent detection and cancer development include HPV type, immune status, multiparity, and tobacco use [3,8,9].

Cervical cancer rates vary significantly in LMICs where access to HPV vaccine and screening remains limited. While high HPV prevalence in the teens and early twenties likely reflects initiation of sexual activity, surveillance in many LMICs shows a second HPV prevalence peak in older women that is unrelated to sexual activity, or a relatively stable prevalence at all ages in some countries in sub-Saharan Africa [10]. One hypothesis for what could be causing higher HPV prevalence in older women in LMICs is loss of immunologic control of HPV infection caused by an immune dysregulating agent. As well-demonstrated by HIV-HPV co-infection and high/early cervical cancer incidence in people living with HIV (PLWH), any agent that reduces the host’s ability to produce a robust Th1 (anti-viral) immune response during early HPV infection theoretically increases the host’s risk of persistent HPV infection and cervical cancer [11,12]. Every effort should be made to identify and eliminate risk factors that permit hrHPV persistence to decrease cervical cancer risk.

Given the geographic overlap between LMICs with high cervical cancer rates and LMICs with high helminth burdens [13,14], it has been hypothesized that the systemic immune dysregulation, as seen with HIV, can also be caused by chronic helminth infection (specifically schistosomiasis/bilharzia, hookworm, *Ascaris,* and *Trichuris*). Even a modest systemic immune dysregulation may facilitate hrHPV persistence and thereby increase cervical cancer risk [8,11,15–17]. Further, chronic helminth infection may trigger direct and indirect changes to local immune microenvironments such as the cervico-vaginal mucosal immune system, changes that could impede hrHPV clearance [11]. Urinary schistosomiasis also may act directly on the cervical epithelia (e.g., local inflammation from schistosomiasis eggs) to make it easier for cervical cancer to develop [17]. Any co-infection which (a) allows HPV to alter the host’s immune response and/or (b) increases local inflammation potentially increases the risk of developing cervical cancer for people with HPV and may lead to earlier onset disease [11].

This scoping review focuses specifically on two research questions: (1) what epidemiological evidence exists describing an association between helminth infection and HPV prevalence, persistence, or progression of dysplasia and (2) what gaps exist in the research? Although basic biology related to this topic has been reviewed elsewhere, this scoping review will summarize epidemiologic research in this area [11]. The findings of this review will further enrich our understanding on helminth infections as risk factors for HPV infections and cervical cancer, providing helpful insights to better inform and strengthen public health programs that seek to facilitate access to deworming treatments and cervical cancer prevention and control programs in LMICs.

## Methods

The scoping review followed established methodological guidelines for scoping reviews [18,19]. Reporting followed the Preferred Reporting Items for Systematic reviews and Meta-Analyses extension for Scoping Reviews (PRISMA-ScR) Checklist (S1 File PRISMA checklist) [20]. The scoping review was conducted in collaboration with an NIH librarian (AL) but did not have a pre-registered protocol. The search strategy and methodology were developed iteratively with expert guidance to ensure comprehensiveness and rigor. The search strategy and all methodological decisions are documented in the manuscript and supplemental resources to ensure transparency and reproducibility.

### Eligibility criteria

We included population and clinical studies (e.g., cohort, case-control, follow-up, observational, cross-sectional, or prospective studies) conducted in LMICs that addressed helminth infections, including schistosomiasis/bilharzia, hookworms, *Ascaris*, and *Trichuris*, and either HPV or cervical cancer. We included original primary research articles, conference abstracts/proceedings, conference papers, books, and book chapters published between 1990 and 2022. We selected 1990 as the cutoff date since this was when high quality HPV (PCR-based) testing was introduced. We included articles written in English or Spanish. We excluded studies conducted in the United States or other upper-middle or high-income countries because helminth infections are not endemic in these countries. We also excluded studies that did not specify the type of helminth studied, reported on animal- or cell-based laboratory studies, or focused on assessing the association between helminth infections and other types of cancers (e.g., bladder cancer). We excluded systematic reviews and meta-analysis, but we scanned the references of relevant reviews to identify other potentially relevant articles. We excluded editorials, letters, and case studies.

### Information sources

We defined our search strategy in collaboration with an NIH biomedical librarian (AL), who conducted the database searches. We conducted the search for studies published after 1990 using five databases: Embase (Elsevier); Global Health (CABI Abstracts); PubMed (National Library of Medicine); Scopus (Elsevier); and Web of Science: Core Collection (Clarivate Analytics).

### Search strategy

Search terms included keywords and controlled vocabulary terms (e.g., MeSH, EMTREE) for each concept of interest (helminths, HPV, cervical cancer, LMICs) (see S2 File for full list of search terms for each database). EndNote 20 (Clarivate Analytics) was used to collect, manage, and identify duplicate records from the literature searches. Additionally, the reference lists of included articles and relevant systematic reviews and meta-analyses were scanned by two reviewers to identify other potentially relevant articles. Any articles identified in this manner were then screened using the procedure below.

### Selection of sources of evidence

Two reviewers (AF and MCM) piloted the screening process on a random sample of records selected by the biomedical librarian. Resulting changes to the screening process and eligibility criteria were completed prior to commencing the official screening steps. The reviewers independently screened each unique record retrieved from the literature searches via a two-step screening process using Covidence (Veritas Health Innovations). First, the reviewers screened all titles and abstracts using the established eligibility criteria. Second, records remaining after title and abstract review underwent full text screening using the same eligibility criteria. The reviewers met weekly to discuss disagreements and reach consensus. When needed, other members of the team informed screening decisions and resolved discrepancies.

### Data charting process

Once the final set of included records was identified, the two reviewers developed a data extraction codebook and piloted it using a randomly selected subset of 15 records. Both reviewers independently collected data using Covidence. Discrepancies between the two reviewers were resolved by a third author (PG).

### Variables

The variables extracted from each manuscript included: title, author, publication date, country and setting of study, study design and objectives, age range of participants, type of exposure studied (i.e., type of helminth), methods to measure exposure status, outcome reported, methods to measure outcome status, methods to measure how exposure and outcome related to one another, main analysis method, primary outcome as related to HPV or cervical cancer and helminth infection, as well as comments, limitations and gaps raised by the study authors. The data extraction codebook and additional details on data collection are available in the supplemental materials (S3 File).

### Synthesis of results

Extracted data were organized by authors AF and PG, who grouped exposures into four categories: schistosomiasis/bilharzia, hookworms, *Ascaris*, and *Trichuris*. Since the outcome for this study (HPV or cervical cancer) was relatively broad, study outcomes were synthetized based on what was specifically reported in each study. Included outcomes were cervical cancer, cervical intraepithelial neoplasia (CIN), high grade squamous intraepithelial lesion (HSIL), and HPV prevalence.

Positive associations were determined based on positive effect estimates reported in the study or calculated by the authors. For some studies, OR and 95% CIs were not directly reported in the articles, but were calculated by authors (AF and PG) using data presented in the publications. Because this was a scoping review, reconstructed 2 × 2 tables were used solely to derive effect estimates and were not consistently available for all included studies.

## Results

### Selection of sources of evidence

**Fig 1:**
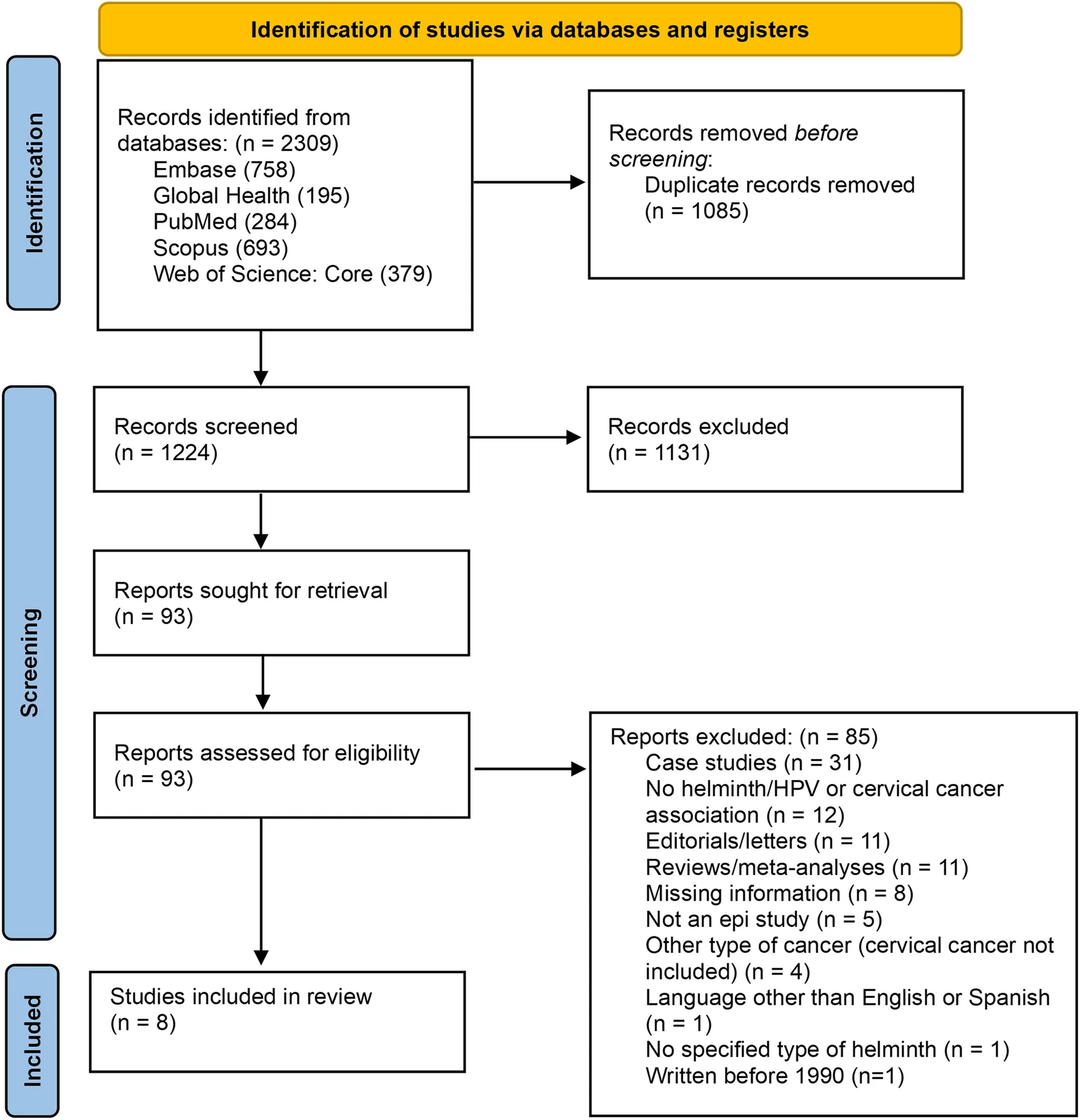
PRISMA flow diagram of the study selection process for the scoping review, showing identification, screening, eligibility assessment, exclusions, and final inclusion of 8 studies.

As shown in Fig 1, 2309 articles were retrieved from the database searches, of which 1085 were duplicates. We screened the titles and abstracts of 1224 articles and, of those, excluded 1131 based on the eligibility criteria. Ninety-three articles that matched eligibility criteria in the title and abstract underwent full text evaluation. Eight underwent final data extraction and were included in this review. Of the 85 excluded, 31 were case studies, 12 did not explore helminth/HPV or cervical cancer association, 11 were editorials or letters, 11 were reviews/meta-analyses, eight were missing information (e.g., details on exposure), five were not epidemiological studies, four discussed cancer other than cervical cancer, one was written before 1990, one did not specify helminth type, and one was in a language other than English or Spanish.

### Characteristics of sources of evidence

Of the eight studies included in this review, six studies were cross-sectional and two were case control (Table 1). Conference abstracts, proceedings, and book chapters were assessed for eligibility. With the exception of the study by Pillay et al. 2017 [21], abstracts were excluded from final analysis due to limited information around helminth/HPV association, description of a case study, or other exclusion reasons as listed in Fig 1. Seven of the eight studies were conducted in Africa, which included five countries. One study was conducted in Peru. See Table 1 for a full breakdown of data characteristics.

**Table 1:** Key characteristics of included studies.

Study	Sample Size	Type of Study	Country	Exposure	Method to Measure Exposure	Outcome	Method to Measure Outcome	Effect Estimate (95% CI)	Association
Gravitt, et. al (2016) [11]	292	Cross-sectional	Peru	Current Hookworm, *Ascaris*, *Trichuris*, or *Strongyloides* infection	Stool sample testing using a direct method, a modified Baerman method, and a modified Ritchie method	HPV prevalence	HPV DNA PCR testing of cervical brush specimens	1.60 (1.0, 2.7)^c^	Positive*
Holali Ameyapoh, et. al (2021) [27]	367	Cross-sectional	Togo	Current Hookworm infection	Urine and stool sample testing using Kato Katz and urine sedimentation methods	HPV prevalence	HPV DNA PCR testing of vaginal and cervical swabs	2.22 (1.32, 3.75)^a^	Positive
Kjetland, et. al (2008) [22]	37	Cross-sectional	Zimbabwe	Current Schistosomiasis infection	Cytology; wet mounts; histology results from biopsies; urine microscopy; a single stool sample was processed by the Kato-Katz technique	HPV prevalence	HPV DNA PCR testing of genital swab	1.9 (1.1, 3.6)^b^	Positive
						High grade squamous intraepithelial lesion (HSIL)		7.1 (0.5, 92.1)^b*^	
Petry, et. al (1995) [25]	138 cases; 35 controls	Case-control	Tanzania	Current Schistosomiasis infection	Urine microscopy; Histology results of biopsies	Cervical Cancer	Visual inspection with acetic acid; Histology results of biopsies; Dot blot hybridization assay of cervical swabs; Cytology; HPV DNA PCR testing of cervical swabs	2.76 (0.9, 8.46)^d^	Positive*
Petry, et. al (2003) [26]	109 patients; 109 controls	Case-control	Tanzania	Current or recent Schistosomiasis infection	Cytology; Histology results of biopsies	Cervical Cancer	Cytology; HPV DNA PCR testing of cervical swabs; Visual Inspection with Acetic Acid; Histology results of biopsies	2.83(0.27,29.9)^d^	Positive*
Pillay, et. al (2017) [21]	833	Cross-sectional	South Africa	Current Schistosomiasis infection	Cytology	Cervical Cancer	Cytology; HPV genotyping	5.6 (1.6, 21.0)^a^	Positive
Rafferty, et. al (2021) [23]	237	Cross-sectional	Zambia	Current Schistosomiasis infection	*Schistosoma* DNA PCR testing of self-sampled genital swabs; Urine circulating anodic antigen testing of urine samples; Urine microscopy; Portable colposcopy of urine samples	Cervical Intraepithelial Neoplasia (CIN)	Visual Inspection with Acetic Acid	6.08 (1.58, 23.37)^b^	Positive
Swanepoel, et. al (2012) [24]	1087	Cross-sectional	South Africa	Current Schistosomiasis infection	Cytology; Histology results of LEEP	HPV prevalence	Cytology; Histology results of LEEP	1.75 (0.58, 5.31)^d^	Positive*

Effect estimates are as reported by each study with a. Crude (unadjusted) odds ratios; b. Adjusted odds ratios; c. Adjusted Prevalence ratios; d. OR and 95% CIs were not directly reported in the papers, they were calculated based on the 2x2 tables that were derived directly from the data reported * Positive trend: indicates an effect estimate greater than the null value but not statistically significant because the 95% confidence interval includes the null value.

### Exposures

Regarding exposure, six studies evaluated participants for schistosomiasis (all in Africa) and two evaluated participants for intestinal helminth infections. Specimens used to evaluate participants for helminth infections varied by study (Table 1). The six studies that evaluated their participants for schistosomiasis did so by either cytology; histology results from tissue removed through loop electrosurgical excision procedure (LEEP) or biopsies; portable colposcopy of urine samples; stool samples processed by the Kato-Katz technique; Schistosoma DNA PCR testing of self-sampled genital swabs; urine circulating anodic antigen testing of urine samples; urine microscopy; or wet mounts. The two studies that evaluated their participants for hookworm did so by either urine and stool sample testing using Kato Katz and urine sedimentation methods. The one study that evaluated their participants for Hookworm, *Ascaris, Trichuris,* or *Strongyloides* infection did so by stool sample testing using a direct method, a modified Baerman method and a modified Ritchie method. See Table 1 for a full breakdown of exposure measurement data.

### Outcomes

The measured outcome was HPV prevalence for four studies, cervical cancer for three, cervical intraepithelial neoplasia (CIN) for one, and high-grade squamous intraepithelial lesion (HSIL) for one. Methods to measure each type of outcome varied by study (Table 1). Of the four studies that evaluated HPV prevalence as the outcome, three used HPV DNA PCR testing and one used cytology and histology results of loop electrosurgical excision procedure (LEEP). Of the three studies that evaluated cervical cancer as the outcome, three used cytology; two used histology results of biopsies; two used HPV DNA PCR testing; two used visual inspection with acetic acid (VIA); one used dot blot hybridization assay of cervical swabs; and one study conducted HPV genotyping. See Table 1 for a full breakdown of outcome measurement data.

### Epidemiologic associations between helminths and hrHPV-related cervical dysplasia or cancer

Schistosomiasis and hrHPV-related cervical dysplasia/cancer – All six studies that explored the relationship between schistosomiasis and cervical neoplasia found a positive association between the two conditions, though most lacked statistical significance. Kjetland et al.’s study [22] in Zimbabwe suggested that women with schistosomiasis were more likely to have hrHPV (adjusted OR: 1.9; 95% CI: 1.1–3.6) as well as HSIL (adjusted OR: 7.1; 95% CI: 0.5-92.1). Although these findings are not statistically significant, they indicate a trend towards a positive association, highlighting the need for robust studies to better understand the association between schistosomiasis, hrHPV infections and HSIL. The Zambian study showed similarly high risk of CIN (adjusted OR: 6.08; 95% CI: 1.58–23.37) for women with schistosomiasis [23]. Swanepoel et al.’s [24] cross-sectional study of South African women with HIV reported that participants with schistosomiasis had 75% higher odds of CIN2+ or cervical cancer compared with women whose biopsies showed CIN1 or koilocytosis (OR: 1.75; 95% CI: 0.58–5.31), though results from this study were not statistically significant (see Table 1 legend for explanation on OR calculation). Pillay et al’s [21] South African study (crude OR: 5.6; 95% CI: 1.6–21.0) found a positive association between schistosomiasis and cervical cancer. Similarly, both Tanzanian studies, the Petry et al. study (1995) [25] (OR: 2.76; 95% CI: 0.9–8.46) and the Petry et al study (2003) [26] (OR: 2.83, 95% CI: 0.27–29.96) found an association between schistosomiasis and cervical cancer that is not statistically significant but suggests a positive trend, warranting more robust studies (see Table 1 legend for explanation on OR calculation).

Intestinal helminth infection and hrHPV-related cervical dysplasia/cancer – Gravitt et al.’s [11] Peruvian study suggests that women with intestinal helminth infection were more likely to be HPV positive (adjusted prevalence ratio [aPR]: 1.6; 95% CI: 1.0–2.7). Holali Ameyapoh et al.’s [27] Togo study similarly showed hookworm infection is associated with HPV infection (OR: 2.22; 95% CI: 1.32–3.75).

## Discussion

The results presented in this study, provide an exploratory review of the epidemiological evidence that could address well-described interactions between parasitic infection and antiviral immune suppression. The published evidence indicates a possible positive association between helminth infections and either cervical HPV persistence or cervical precancerous dysplasia, but not invasive cervical cancer. This conclusion is supported by the epidemiologic data consistent with helminths playing a role in the immune dysregulation of the oncogenic virus HPV instead of playing a role as a direct carcinogen for cervical cancer (e.g., as is the case for schistosomiasis and bladder cancer) [11]. Although most effect estimates (5 of the 8 studies) did not reach statistical significance, the direction of the association was positive across the included studies. The lack of statistical significance may reflect multiple factors, including limited sample sizes, residual confounding, selection bias, measurement error, and the observational nature of the included studies. In addition, several studies were not primarily designed to evaluate the association between helminth infections and HPV-related outcomes. Therefore, larger, well-designed studies specifically powered to investigate these associations are warranted. Since the data suggests a positive trend towards the association between helminth infection and cervical intraepithelial neoplasia and given the high burden of helminth and HPV co-infection in LMICs, whose populations bear 85% of the global cervical cancer burden, further evaluation of this potential interaction is warranted.

Although these studies do not provide evidence of helminth infection as a direct carcinogen independent of HPV, authors suggest the potential link between schistosomiasis infection and DNA damage which accelerates carcinogenesis of hrHPV, so this mechanism cannot be ruled out from these studies [28]. Another hypothesis is that helminths work upstream in the causal pathway by increasing risk of persistent HPV detection by activating the T helper type 2 immune response, which in turn downregulates the T helper type 1 response needed to control viral infections like HPV [8]. Some hypothesize that schistosomiasis can lead to HPV persistence through damage to the cervical epithelium and an additional DNA damage inflammation carcinogenic effect [28]. Additionally, schistosomiasis could cause local immune modulation, providing a tolerogenic environment for HPV to persist [28]. While more research is needed to test these hypotheses, known possible mechanisms for the association give important biological credibility to the findings.

This study has several limitations. Amongst the scope of this review, there is a likelihood of positive reporting bias, therefore there may be a lack of publications on studies that did not find an association. Additionally, none of the studies included adjusted for important confounding effects of sexual behaviors, HIV status, and other immune suppressive infections. To the extent that these exposures were differential between our helminth exposure groups, the point estimates may be biased. Our search for literature used five major international databases (PubMed, Web of Science, Scopus, Embase, and Global Health) that provide comprehensive coverage of peer-reviewed literature; however, a limitation of our search strategy is the exclusion of regional databases specifically designed to index research from Latin America (SciELO, LILACS), Africa (AJOL), and Asia (WPRIM). Hence, studies published in local or regional journals not indexed in major international databases may have been missed. Future reviews would benefit from incorporating these regional databases to ensure more complete global representation of the evidence base.

The inclusion of papers written only in English or Spanish is another limitation of this review as it introduces substantial language bias and may marginalize important scientific contributions from non-English speaking researchers. By excluding publications in other languages (e.g., French and Portuguese) and regional or local journals, we likely missed data from regions where both helminth infections and HPV are endemic and may be well-studied. This language restriction was implemented due to the practical constraints of requiring translation services and multilingual reviewers, as well as concerns about maintaining consistency in data extraction and quality assessment across languages. However, we acknowledge that this pragmatic decision significantly limits the comprehensiveness and our ability to draw conclusions that represent global evidence. Any data from studies published before 1990 that could be relevant to this topic was not looked at and furthermore, studies published in local journals and not indexed in the journals included in this review could have been missed. As the search for this scoping review was conducted in April 2022, we might have missed some recent publications or grey literature published since then.

### Public health impact

The World Health Organization’s road map to eliminate neglected tropical diseases (NTDs) by 2030 outlines strategies to prevent, control, and eliminate NTDs, including helminth infection [29]. Strategies include improvements in water, sanitation, and hygiene (WASH), innovation in diagnostics, and increased access to deworming treatments [29]. Recognizing the overlap between hrHPV and helminth infections, public health programs that bundle cervical cancer and helminth prevention and control programs could have significant impacts. Efforts to understand the interaction between helminth infection, persistence of hrHPV, and cervical cancer, are important to increase the evidence-base for these public health programs and improve health outcomes in the population [8,11].

## Conclusion

Considering this high prevalence and the overlap of HPV and helminth infection, and the impact these infections have on the quality of life of populations in LMICs, studies that help expand our understanding on the epidemiological evidence and health impact of these infections are essential. The associations identified through this scoping review contribute towards the evidence-base that can be helpful to inform public health programs and guidelines. Since helminth re-infection is common in endemic areas, evidence-based prevention and control measures, including preventive chemotherapy implemented according to WHO recommendations and improvements in water, sanitation, and hygiene (WASH), are important strategies to reduce transmission and reinfection. It is then important to consider the syndemics of helminths and HPV infections (and likely many other carcinogenic viral infections) and how they change the individual and population ecology, especially in rural areas where cervical cancer is high [30]. Given major public health initiatives for NTDs and HPV/cervical cancer, we have a time-sensitive opportunity for cross-disciplinary collaboration to more fully understand the relationships between these infections and their causal pathways as well as the biological mechanisms of interactions to enable the identification of effective public health interventions.

## Supporting information

S1 File PRISMA-ScR Checklist. Completed Preferred Reporting Items for Systematic Reviews and Meta-Analyses Extension for Scoping Reviews (PRISMA-ScR) Checklist. From: Tricco AC, Lillie E, Zarin W, O’Brien KK, Colquhoun H, Levac D, et al. PRISMA Extension for Scoping Reviews (PRISMAScR): Checklist and Explanation. Ann Intern Med. 2018;169:467–473. https://doi.org/10.7326/M18-0850.
(DOCX)

S2 File Search strategy and databases searched. Complete electronic search strategies, database-specific search syntax, search dates, database coverage, and applied limits and filters for all databases included in the review.
(DOCX)

S3 File Data extraction codebook. Codebook describing the review objective, eligibility framework, variables extracted from each study, and data extraction fields used during the review.
(DOCX)

## Abbreviations

CI: confidence interval
CIN: cervical intraepithelial neoplasia
DNA: deoxyribonucleic acid
HIV: human immunodeficiency virus
HPV: human papillomavirus
hrHPV: high-risk human papillomavirus
HSIL: high-grade squamous intraepithelial lesion
LEEP: loop electrosurgical excision procedure
LMICs: low- and middle-income countries
NIH: National Institutes of Health
NTDs: neglected tropical diseases
OR: odds ratio
PCR: polymerase chain reaction
PLWH: people living with HIV
PRISMA-ScR: Preferred Reporting Items for Systematic reviews and Meta-Analyses extension for Scoping Reviews
Th1: T helper 1 cells
VIA: visual inspection with acetic acid
WASH: water, Sanitation, and Hygiene
