# Supplementary material for: Understanding the association between immune-modulating helminths and HPV or cervical cancer: A scoping review
Source: PLoS Negl Trop Dis. 2026 Sep 2;20(9):e0013308. doi: 10.1371/journal.pntd.0013308 (PMC13537589; doi:10.1371/journal.pntd.0013308)
Supplement: Supplementary file 4 [file pntd.0013308.s005.docx]

**Comments**

**Minor comments**

- The figure caption of Figure-1 is almost a paragraph. Make it short and precise.
- Re-arrange your abbreviations on page-19 in alphabetical order

**Specific comments**

Page-5

L_95_: insert the word “enrich” between ‘further’ and ‘our’

**Results**

Page-12

L_248_ – L_249_ – write the full form of CIN and HSIL with the abbreviated for the first time and then you can use the abbreviated form only. The same comment holds true for LEEP in L_251_

Page-13

L_273_: delete the letter “a” found between the words ‘but’ and ‘suggests’

**Discussion**

Page-18

L_343_: delete “HrHPV” and replace it by “hrHPV”

**Comments on references**

1. The number of authors in your 10^th^ reference is 7. Therefore, delete the 7^th^ and replace it by *et al*. The same comment holds true for your 13^th^, 16^th^, 25^th^, 26^th^, references.
2. Your 11^th^ reference lacks page number; include the page number. The same comment holds true for your 19^th^ reference. The same comment holds true for your 20^th^, references.
3. Since your 24^th^ reference is published in a non-periodically publishing journal, delete the initial page number “691” and replace it by the article URL.
