# Supplementary material for: Understanding the association between immune-modulating helminths and HPV or cervical cancer: A scoping review
Source: PLoS Negl Trop Dis. 2026 Sep 2;20(9):e0013308. doi: 10.1371/journal.pntd.0013308 (PMC13537589; doi:10.1371/journal.pntd.0013308)
Supplement: Supplementary file 5 [file pntd.0013308.s006.docx]

**March 6, 2026**

Aysegul Taylan Ozkan, M.D., Ph.D. - Academic Editor

Krystyna Cwiklinski - Section Editor

Shaden Kamhawi - co-Editor-in-Chief

Paul Brindley - co-Editor-in-Chief

*PLOS Neglected Tropical Diseases*

**Subject: Response to Reviewers for Manuscript PNTD-D-25-01055**

Dear Editors,

Thank you for the opportunity to revise our manuscript, "Understanding the association between immune modulating helminths and HPV or cervical cancer: a scoping review." We also wish to extend our gratitude to the reviewers for their insightful and constructive feedback. We have carefully considered all comments and believe that addressing them has significantly strengthened our paper.

Below, we provide a point-by-point response to the issues raised by the reviewers.

**Reviewer #1**

- **Comment:** Methods part of abstract lacks the following description: Selection criteria for the articles, search ‘terms’ for selecting the articles, how many articles were found and how many of them were selected based on the criteria.
  - **Response:** We have updated the abstract to include this information.
- **Comment:** Introduction, from page 4 L70 – L75: The two sentences require reference; L78 – L81: Similarly, requires reference; L87 – L89: Also requires reference.
  - **Response:** We have added the appropriate references in the indicated sections.
- **Comment:** L298: Add the words “the indexers” in the word between ‘in’ and ‘the’.
  - **Response:** We did not fully understand this comment and were therefore unable to make a change. We would be grateful for clarification if the reviewer feels this is a critical point.
- **Comment:** Complete the following sections after conclusion: Acknowledgements; abbreviation; Authors’ contributions.
  - **Response:** These sections have been finalized and included in the revised manuscript.

**Reviewer #2**

- **Comment:** Among the variables extracted from each manuscript, it could have been interesting to extract (if available) HIV status as this could eventually be a confounding bias, especially that almost all studies (7/8) where performed in Africa.
  - **Response:** We thank the reviewer for the comment. Information on HIV status was not reported in the articles included. We have added content to the discussion section regarding HIV status as a potential confounding bias.
- **Comment: Line 229:** are there two studies that evaluated patients for "just hookworm"? (It seems there are 6 schists / 1 "just hookworm" / 1 helminth infections).
  - **Response:** This was a typo and has been corrected in the manuscript**.**
- **Comment:** In table 1, association should not be marked as positive if the result is not statistically significant (consider "not significant, but positive trend").
  - **Response:** We thank the reviewer for this suggestion. We have modified the phrasing to "non-statistically significant but consistent positive trend, warranting more robust studies."

**Reviewer #3**

- **Comment:** Lines 106–108: Please clarify whether the scoping review protocol was published in a scientific repository (OSF, Research Registry, INPLASY, etc.).
  - **Response:** We thank the reviewer for the comment. The scoping review protocol was not published in a repository. However, the review was conducted in collaboration with an NIH librarian (and co-author on this manuscript), and the search strategy and methodology were developed iteratively with expert guidance. All methodological decisions are documented in the manuscript and supplemental materials to ensure transparency and reproducibility.
- **Comment:** A major limitation is the exclusion of databases from endemic regions. Latin America (SciELO, LILACS), Africa (AJOL), and Asia (WPRIM) might contain studies that are otherwise missed. The omission should be justified.
  - **Response:** We thank the reviewer for this point. Our search strategy focused on five major international databases for comprehensive coverage. We acknowledge that the omission of regional databases is a notable limitation and have stated this in the manuscript. We are considering a follow-up review that will include these databases.
- **Comment:** Specify whether the ORs in the text and Table 1 are crude or adjusted.
  - **Response:** We have clarified in the text and in Table 1 whether the reported ORs are crude or adjusted.
- **Comment: Line 249:** The reported CI seems to have a typo... The OR suggests an association between HSIL and schistosomiasis, but it is not statistically significant. Please double-check.
  - **Response:** This has been corrected in the text.
- **Comment: Line 255–256:** Both Petry et al. studies showed positive associations, but they were not statistically significant. Clarify.
  - **Response:** This point has been clarified and corrected in the text.
- **Comment:** Line 266: Out of nine ORs presented, five were not statistically significant. Therefore, it is not appropriate to conclude consistent positive associations; at best, there is a suggestive trend.
  - **Response:** We agree and have adjusted our language. The point estimates were consistently positive despite some being non-statistically significant, likely due to small sample sizes. We have revised our conclusion to state that this trend suggests value in conducting larger, more rigorous studies.
- **Comment**: Line 269: The conclusion should be stated more carefully. Without quality/risk of bias assessment, knowledge of whether ORs are crude/adjusted, and other variables (e.g., HPV vaccination status), the data cannot support a strong hypothesis.
  - **Response:** We acknowledge the reviewer's point. As this is a scoping review, a formal quality/risk of bias assessment is not mandatory. The primary goal was to map existing knowledge and identify research gaps. We have revised the manuscript to emphasize the exploratory nature of this review and to clarify that the observed trend, while not always statistically significant, calls for more rigorous studies.
- **Comment:** The authors should explicitly address whether there is evidence that treating schistosomiasis improves HPV-related lesion outcomes.
  - **Response:** We thank the reviewer for this important question. Treatment outcomes were not an aim of the papers included in our review, and this data was not available. We agree that our findings suggest a positive trend but are far from definitive. Our analysis was intended to review the epidemiological evidence supporting the well-described interactions between parasitic infection and antiviral immune suppression. We conclude that the published evidence indicates a possible positive association that requires more rigorous evaluation.
- **Comment:** Figure 1: Comparing 2016 DALYs vs. 2020 incidence is not appropriate.
  - **Response:** We thank the reviewer for this observation. Our analysis is based primarily on basic research demonstrating the tendency for helminths to induce reactivation of latent DNA viruses, with geographical comparisons serving only as a crude measure of correlation. We have removed Figure 1 from the manuscript.

**Reviewer #4**

- **Comment:** The introduction outlines two questions (epidemiological evidence + gaps), but these are not consistently revisited in the Results and Discussion.
  - **Response:** We thank the reviewer for this comment. We have added content and rephrased sections in the Results and Discussion to more clearly revisit and emphasize these two guiding questions.
- **Comment:** Only English/Spanish studies were included: potential language bias. This should be emphasized more strongly in the limitations**.**
  - **Response:** We thank the reviewer and acknowledge this important limitation. We have strengthened the discussion of this language bias in the limitations section, explaining that the restriction was due to practical constraints but acknowledging its impact on the comprehensiveness of the review.
- **Comment:** The methods state that conference abstracts, proceedings, and book chapters were eligible, but none appear in the included studies. Clarify whether any were retrieved and screened, or whether all excluded.
  - **Response:** We have clarified in the manuscript that only one abstract fulfilled the eligibility criteria and was included in the study.
- **Comment:** While the manuscript suggests schistosomiasis screening in gynecological exams, this recommendation may be premature... Authors should reframe as a potential avenue for research/pilot programs, not a definitive recommendation.
  - **Response:** We thank the reviewer for this valuable feedback. We agree that the recommendation was premature and have removed it from the manuscript, reframing it as a call for further research.
- **Comment:** My suggestion is that the author should add a table summarizing key limitations and potential biases of each included study.
  - **Response:** We thank the reviewer for this suggestion. While we did not formally code for the limitations and biases of each individual study, we have discussed the key limitations of the overall sample of included studies within the manuscript.
- **Comment:** The Results emphasize “consistent positive associations,” but many effect estimates have very wide CIs crossing unity... Overstatement of consistency should be tempered.
  - **Response:** We agree with the reviewer. We have adjusted the language in the results and discussion to temper our claims and more accurately reflect the data, noting the wide confidence intervals and instances of non-significance.

**Reviewer #5**

- **Comment:** The analysis can be further enriched by the inclusion of information regarding other factors such as age of the participants, that may influence susceptibility to chronic HPV infection and cervical cancer, in these studies**.**
  - **Response:** We agree this would be a valuable addition. However, the data in the included studies were often not reported in a manner that allowed for stratification by age, particularly given the small sample sizes. We acknowledge that the evidence base is limited, yet we highlight that the trends point in a positive direction consistent with the basic science.
- **Comment:** More attention should be paid on the possible immunologic mechanisms (with support of a figure) behind the association... I also wonder if it is possible to compare persistent HPV infection and/or cervical cancer rates between regions with high and low helminth prevalence, by using WHO data.
  - **Response:** We thank the reviewer for these excellent suggestions. The immunological mechanisms have been reviewed extensively elsewhere by two of the co-authors (Patti Gravitt and Eva Clark). An analysis using WHO data to compare regional prevalence rates is a wonderful idea but was beyond the scope of the current study.

We thank you again for your time and for the insightful comments, which have helped us improve the quality and clarity of our manuscript. We look forward to your decision.

Sincerely,

Margarita Correa-Mendez

**Corresponding Author**
