# Supplementary material for: Understanding the association between immune-modulating helminths and HPV or cervical cancer: A scoping review
Source: PLoS Negl Trop Dis. 2026 Sep 2;20(9):e0013308. doi: 10.1371/journal.pntd.0013308 (PMC13537589; doi:10.1371/journal.pntd.0013308)
Supplement: Supplementary file 6 [file pntd.0013308.s007.docx]

**June 30, 2026**

Aysegul Taylan Ozkan, M.D., Ph.D. - Academic Editor

Krystyna Cwiklinski - Section Editor

Shaden Kamhawi - co-Editor-in-Chief

Paul Brindley - co-Editor-in-Chief

*PLOS Neglected Tropical Diseases*

**Subject: Response to Reviewers for Manuscript PNTD-D-25-01055**

Dear Editor,

We thank you and the reviewer for the careful evaluation of our manuscript, *“Understanding the association between immune modulating helminths and HPV or cervical cancer: a scoping review.”* We appreciate the suggestions which have helped improve the clarity and quality of the manuscript.

We have revised the manuscript accordingly and provide a point-by-point response below. All changes have been incorporated into the revised manuscript with tracked changes.

**Reviewer Comments and Responses**

**Comment:** The figure caption of Figure 1 is almost a paragraph. Make it short and precise.

**Response:** Thank you for this suggestion. The Figure 1 caption has been revised and shortened for clarity and conciseness. It now reads:

*"Figure 1. PRISMA flow diagram of the study selection process for the scoping review, showing identification, screening, eligibility assessment, exclusions, and final inclusion of 8 studies."*

**Comment:** Re-arrange your abbreviations on page 19 in alphabetical order.

**Response:** Thank you for this observation. The abbreviations list has been revised and reordered alphabetically.

**Specific Comments**

**Page 5**

**Comment:** L95: insert the word “enrich” between ‘further’ and ‘our’.

**Response:** Thank you, the revision has been made.

**Results**

**Page 12**

**Comment:** L248–L249: write the full form of CIN and HSIL with the abbreviation for the first time and then use the abbreviation only. The same comment holds true for LEEP in L251.

**Response:** The full terms for cervical intraepithelial neoplasia (CIN), high-grade squamous intraepithelial lesion (HSIL), and loop electrosurgical excision procedure (LEEP) have been provided at first mention, and the abbreviations are used thereafter.

**Page 13**

**Comment:** L273: delete the letter “a” found between the words ‘but’ and ‘suggests’.

**Response:** Thank you. The typographical error has been corrected.

**Discussion**

**Page 18**

**Comment:** L343: delete “HrHPV” and replace it by “hrHPV”.

**Response:** This has been corrected and revised throughout the manuscript to ensure consistency in terminology.

**Comments on References**

**General Response:** We further revised the bibliography to include the PMID if available and the DOI links. We did this as several cited articles do not have traditional page numbers and may only have an article identifier. Using the [Citing Medicine](https://www.nlm.nih.gov/bsd/uniform_requirements.html#journals) webpage as a guide, we incorporated the PMID and DOI as per items #36.

**Comment 1**

**Comment:** The number of authors in your 10th reference is 7. Therefore, delete the 7th and replace it by et al. The same comment holds true for your 13th, 16th, 25th, and 26th references.

**Response:** Thank you for pointing this out. We changed the citation format to only list the first 6 authors and then include et al. when there is more than 6 authors.

**Comment 2**

**Comment:** Your 11th reference lacks page number; include the page number. The same comment holds true for your 19th and 20th references.

**Response:** Thank you for this observation. These journals do not issue page numbers and only include an article identifier. As described above, we incorporated the PMID and DOI instead as persistent identifiers for the articles.

**Comment 3**

**Comment:** Since your 24th reference is published in a non-periodically publishing journal, delete the initial page number “691” and replace it by the article URL.

**Response:** Thank you for your comment. Response: Like the above comment, this article does not have page numbers and only has an article identifier. We included the DOI and PMID for the article.

**Reviewer #3**

**Comment:** Prefer “immunomodulatory” or “immune-modulating” instead of “immune modulating” in the title and running text.

**Response:** Thank you. We have revised the title and manuscript text to use “immune-modulating” consistently throughout the manuscript.

**Comment:** Line 166: Change to synthesized.

**Response:** The term has been corrected to “synthesized.”

**Comment:** Line 259: The authors stated: “…though some lacked statistical significance”. However, most (5 out of eight studies) lacked statistical significance.

**Response:** Thank you for this observation. The text has been revised to indicate that most (5 of the 8 studies) did not reach statistical significance while maintaining that the direction of association was consistently positive across the included studies.

**Comment:** Lines 265–268: The authors cited Swanepoel et al. study. It is important to mention that the result from this study was not statistically significant.

**Response:** We have revised the text to explicitly state that the findings reported by Swanepoel et al. were not statistically significant.

**Comment:** Line 271: Fix the CI. The lower confidence bound is incorrect.

**Response:** The confidence interval has been corrected.

**Comment:** Lines 276–278: The confidence interval contains the null value (1.0). Therefore, change the verb “demonstrate” to “suggest.” Additionally, Table 1 states that the effect measurement used was a prevalence ratio while the text says OR. Clarify it.

**Response:** We revised the text to use more cautious language (“suggest”) and corrected the description of the effect estimate to indicate that the study reported an adjusted prevalence ratio rather than an odds ratio, consistent with Table 1.

**Comment:** Line 293: The authors stated: “Although not all effect measurements were statistically significant…”. To be more precise indicate that most effect measures (5 out of 8 studies) were not statistically significant.

**Response:** We have revised the statement to indicate that most effect estimates (5 of the 8 studies) did not reach statistical significance.

**Comment:** Lines 303–305: They might not be statistically significant exclusively due to a small sample size. Did all the included studies calculate a sample size? Were the sample sizes inadequate to test the current study hypothesis? Additionally, take into account that the results might be biased due to the observational nature of the included studies and that those studies could have other main outcomes not related to this scoping review.

**Response:** Thank you for this important comment. We agree that the lack of statistical significance cannot be attributed solely to sample size limitations. As this was a scoping review, we did not systematically assess whether individual studies conducted sample size calculations or were adequately powered to evaluate the associations of interest. We revised the Discussion to acknowledge that the findings may also reflect residual confounding, selection bias, measurement error, the observational nature of the included studies, and the fact that several studies were not primarily designed to evaluate associations between helminth infections and HPV-related outcomes.

**Comment:** Line 343: Use “hrHPV” consistently.

**Response:** The manuscript has been revised to use “hrHPV” consistently throughout.

**Comment:** Lines 353–356: The authors recommend frequent (every three months) deworming of reproductive-age individuals. The proposed frequency is not consistent with WHO recommendations.

**Response:** Thank you for this important observation. We agree that the statement was overly prescriptive and not fully aligned with current WHO preventive chemotherapy recommendations. The text has been revised to remove the specific deworming frequency and instead refer to evidence-based prevention and control measures, including preventive chemotherapy implemented according to WHO recommendations and improvements in water, sanitation, and hygiene (WASH).

**Comment:** Change to deworming.

**Response:** Thank you for this observation. The term “de-worming” has been removed as part of revisions made in response to comments regarding alignment with current WHO preventive chemotherapy recommendations. The revised text now refers more broadly to evidence-based helminth prevention and control strategies.

**Comment:** Prefer “antiviral” over “anti-viral.”

**Response:** The manuscript has been revised accordingly to use antiviral.

**Comment:** Add missing abbreviations in the abbreviations list: PLWH, NTDs, WASH.

**Response:** These abbreviations have been added to the abbreviations list.

**Comment:** Table 1: Not all associations can be interpreted as positive using an asterisk. Either some are positive and the rest are null, or all are positive and only those which confidence intervals include the null value should include the asterisk.

**Response:** Thank you for this observation. We agree that the previous use of the asterisk in Table 1 could be misinterpreted. The table has been revised so that the asterisk is applied only to positive associations whose confidence intervals include the null value and therefore did not reach statistical significance. The corresponding table footnote has also been revised to clarify this distinction.

**Comment:** Consider adding the extracted 2×2 tables from studies that did not report effect measurements (these should be placed in Supplemental Information).

**Response:** Thank you for this suggestion. We agree that providing the underlying 2×2 tables could enhance transparency. However, as this was a scoping review, our objective was to summarize and synthesize the effect estimates reported in, or derived from, the studies included rather than to perform a de novo quantitative reanalysis. We have clarified in the Methods section that, for studies that did not report effect estimates directly, odds ratios and confidence intervals were derived from data reported in the original publications when sufficient information was available. Reconstructed 2×2 tables were not consistently available for all included studies.

We thank the reviewers and editor for their thorough review and comments and believe that the revisions have strengthened the manuscript. We hope the revised version is now suitable for publication in *PLOS Neglected Tropical Diseases*.

Sincerely,

Margarita Correa-Méndez, on behalf of all authors
