# Supplementary material for: Understanding the association between immune-modulating helminths and HPV or cervical cancer: A scoping review
Source: PLoS Negl Trop Dis. 2026 Sep 2;20(9):e0013308. doi: 10.1371/journal.pntd.0013308 (PMC13537589; doi:10.1371/journal.pntd.0013308)
Supplement: Supplementary file 7 [file pntd.0013308.s008.docx]

**July 22, 2026**

**Dr. Krystyna Cwiklinski**
Section Editor
PLOS Neglected Tropical Diseases

**Dr. Shaden Kamhawi**
Co-Editor-in-Chief
PLOS Neglected Tropical Diseases

**Dr. Paul Brindley**
Co-Editor-in-Chief
PLOS Neglected Tropical Diseases

**Subject: Response to Reviewers for Manuscript PNTD-D-25-01055**

Dear Editors and Reviewers,

We sincerely thank the Editor and Reviewers for their thoughtful evaluation of our manuscript and their constructive feedback, which has helped improve its quality, clarity, and accuracy. We greatly appreciate the time and effort devoted to the review process.

We also apologize that some revisions may not have been adequately reflected in our previous submission, resulting in some comments remaining unaddressed. We carefully reviewed all reviewer and editorial comments again and revised the manuscript accordingly. We believe all comments have now been fully addressed and that the manuscript has been substantially strengthened.

Below, we provide a point-by-point response describing how each comment has been addressed.

**Reviewer #3**

We thank Reviewer #3 for the positive assessment of our revised manuscript and for recognizing the improvements made in response to the previous review. We also appreciate the additional editorial suggestions, which have been addressed as follows:

- **Title and Short Title:** Corrected the term *immune-modulating* in both the full and short titles.
- **Consistency of Librarian Initials:** Standardized the librarian's initials throughout the manuscript.
- **Abbreviations:** Updated the abbreviations list to include PLWH (people living with HIV), NTDs (neglected tropical diseases), and WASH (water, sanitation, and hygiene), and reviewed the list for consistency.
- **Table 1 Asterisk Notation:** Revised the asterisk notation so that it is used only for positive associations whose 95% confidence intervals include the null value and therefore did not reach statistical significance. The table legend has been retained to explain the notation.

**Minor Editorial Comments**

All editorial comments have been addressed, including:

- Shortening the Figure 1 caption.
- Adding the word *enrich* in the Introduction as suggested.
- Defining cervical intraepithelial neoplasia (CIN), high-grade squamous intraepithelial lesion (HSIL), and loop electrosurgical excision procedure (LEEP) at first mention.
- Correcting typographical and formatting inconsistencies, including removal of the extraneous "a" in the Results section and standardization of *hrHPV* capitalization.
- Updating the reference list to conform to journal style, including author listings, page information, and article identifiers where appropriate.

**Data Availability and Reproducibility**

The submitted manuscript and supplemental materials include the information requested regarding transparency and reproducibility. Specifically, they provide details on study selection, extracted data, the search strategy, screening process, data extraction methods, and other methodological information necessary to support reproducibility of the review.

Once again, we thank the Editor and Reviewers for their careful and constructive feedback. We believe these revisions have strengthened the manuscript and hope the revised version is now suitable for publication.

Sincerely,

Margarita Correa-Méndez, on behalf of all authors
