# Supplementary material for: Understanding the association between immune-modulating helminths and HPV or cervical cancer: A scoping review
Source: PLoS Negl Trop Dis. 2026 Sep 2;20(9):e0013308. doi: 10.1371/journal.pntd.0013308 (PMC13537589; doi:10.1371/journal.pntd.0013308)
Supplement: Peer review history

# Peer review: Decision Letter 0

**DOI:** 10.1371/journal.pntd.0013308.r001

**Authors:** Aysegul Taylan Ozkan, Krystyna Cwiklinski

8 Sep 2025

Understanding the association between immune modulating helminths and HPV or cervical cancer: a scoping review

PLOS Neglected Tropical Diseases

Dear Dr. Correa-Méndez,

Thank you for submitting your manuscript to PLOS Neglected Tropical Diseases. After careful consideration, we feel that it has merit but does not fully meet PLOS Neglected Tropical Diseases's publication criteria as it currently stands. Therefore, we invite you to submit a revised version of the manuscript that addresses the points raised during the review process.

Please submit your revised manuscript within 60 days Nov 07 2025 11:59PM. If you will need more time than this to complete your revisions, please reply to this message or contact the journal office at plosntds@plos.org. When you're ready to submit your revision, log on to https://www.editorialmanager.com/pntd/ and select the 'Submissions Needing Revision' folder to locate your manuscript file.

Please include the following items when submitting your revised manuscript:

* A rebuttal letter that responds to each point raised by the editor and reviewer(s). You should upload this letter as a separate file labeled 'Response to Reviewers'. This file does not need to include responses to any formatting updates and technical items listed in the 'Journal Requirements' section below.

* A marked-up copy of your manuscript that highlights changes made to the original version. You should upload this as a separate file labeled 'Revised Manuscript with Track Changes'.

* An unmarked version of your revised paper without tracked changes. You should upload this as a separate file labeled 'Manuscript'.

If you would like to make changes to your financial disclosure, competing interests statement, or data availability statement, please make these updates within the submission form at the time of resubmission. Guidelines for resubmitting your figure files are available below the reviewer comments at the end of this letter.

We look forward to receiving your revised manuscript.

Kind regards,

Aysegul Taylan Ozkan, M.D., Ph.D.,

Academic Editor

PLOS Neglected Tropical Diseases

Krystyna Cwiklinski

Section Editor

PLOS Neglected Tropical Diseases

Shaden Kamhawi

co-Editor-in-Chief

PLOS Neglected Tropical Diseases

orcid.org/0000-0003-4304-636XX

Paul Brindley

co-Editor-in-Chief

PLOS Neglected Tropical Diseases

orcid.org/0000-0003-1765-0002

**Journal Requirements:**

1) Please ensure that the CRediT author contributions listed for every co-author are completed accurately and in full.

At this stage, the following Authors/Authors require contributions: Allison Frank, Margarita Correa-Méndez, Alicia A. Livinski, Kalina Duncan, Eva H Clark, and Patti G Gravitt. Please ensure that the full contributions of each author are acknowledged in the "Add/Edit/Remove Authors" section of our submission form.

The list of CRediT author contributions may be found here: https://journals.plos.org/plosntds/s/authorship#loc-author-contributions

2) Please ensure that the Title in your manuscript file and the Title provided in your online submission form are exactly the same.

3) Please upload all main figures as separate Figure files in .tif or .eps format. For more information about how to convert and format your figure files please see our guidelines:

https://journals.plos.org/plosntds/s/figures

4) We have noticed that you have uploaded Supporting Information files, but you have not included a list of legends. Please add a full list of legends for your Supporting Information files after the references list.

5) Some material included in your submission may be copyrighted. According to PLOSu2019s copyright policy, authors who use figures or other material (e.g., graphics, clipart, maps) from another author or copyright holder must demonstrate or obtain permission to publish this material under the Creative Commons Attribution 4.0 International (CC BY 4.0) License used by PLOS journals. Please closely review the details of PLOSu2019s copyright requirements here: PLOS Licenses and Copyright. If you need to request permissions from a copyright holder, you may use PLOS's Copyright Content Permission form.

Please respond directly to this email and provide any known details concerning your material's license terms and permissions required for reuse, even if you have not yet obtained copyright permissions or are unsure of your material's copyright compatibility. Once you have responded and addressed all other outstanding technical requirements, you may resubmit your manuscript within Editorial Manager.

Potential Copyright Issues:

i) Figure 1. Please (a) provide a direct link to the base layer of the map (i.e., the country or region border shape) and ensure this is also included in the figure legend; and (b) provide a link to the terms of use / license information for the base layer image or shapefile. We cannot publish proprietary or copyrighted maps (e.g. Google Maps, Mapquest) and the terms of use for your map base layer must be compatible with our CC BY 4.0 license.

Note: if you created the map in a software program like R or ArcGIS, please locate and indicate the source of the basemap shapefile onto which data has been plotted.

If your map was obtained from a copyrighted source please amend the figure so that the base map used is from an openly available source. Alternatively, please provide explicit written permission from the copyright holder granting you the right to publish the material under our CC BY 4.0 license.

If you are unsure whether you can use a map or not, please do reach out and we will be able to help you. The following websites are good examples of where you can source open access or public domain maps:

* U.S. Geological Survey (USGS) - All maps are in the public domain. (http://www.usgs.gov)

* PlaniGlobe - All maps are published under a Creative Commons license so please cite u201cPlaniGlobe, http://www.planiglobe.com, CC BY 2.0u201d in the image credit after the caption. (http://www.planiglobe.com/?lang=enl)

* Natural Earth - All maps are public domain. (http://www.naturalearthdata.com/about/terms-of-use/).

6) Thank you for stating " no competing interests exist." Please revise your current Competing Interest statement to the standard "The authors have declared that no competing interests exist."

7) Please ensure that the PRISMA flowchart describing the included/excluded literature is uploaded as Figure 1. Information about the PRISMA guidance, and blank flowcharts and checklists, can be found here: http://www.prisma-statement.org/.

Note: If the reviewer comments include a recommendation to cite specific previously published works, please review and evaluate these publications to determine whether they are relevant and should be cited. There is no requirement to cite these works unless the editor has indicated otherwise.

**Reviewers' Comments:**

Reviewer's Responses to Questions

**Key Review Criteria Required for Acceptance?**

As you describe the new analyses required for acceptance, please consider the following:

**Methods**

-Are the objectives of the study clearly articulated with a clear testable hypothesis stated?

-Is the study design appropriate to address the stated objectives?

-Is the population clearly described and appropriate for the hypothesis being tested?

-Is the sample size sufficient to ensure adequate power to address the hypothesis being tested?

-Were correct statistical analysis used to support conclusions?

-Are there concerns about ethical or regulatory requirements being met?

Reviewer #1: All the above do not holds true, as the manuscript is a review

Reviewer #2: - The objectives are clearly stated and scientifically relevant.

- The study design is appropriate and methods are well described.

- Among the variables extracted from each manuscript, it could have been interesting to extract (if available) HIV status as this could eventually be a confounding biais, especially that almost all studies (7/8) where performed in Africa.

Reviewer #3: Lines 106–108: Please clarify whether the scoping review protocol was published in a scientific repository (OSF, Research Registry, INPLASY, etc.). Registration is important to ensure that the authors followed a pre-established protocol and avoided major deviations.

A major limitation is the exclusion of databases from endemic regions. Latin America (SciELO, LILACS), Africa (AJOL), and Asia (WPRIM) might contain studies that are otherwise missed. The omission should be justified.

Reviewer #4: The introduction outlines two questions (epidemiological evidence + gaps), but these are not consistently revisited in the Results and Discussion.

Only English/Spanish studies were included: potential language bias. This should be emphasized more strongly in the limitations.

The methods state that conference abstracts, proceedings, and book chapters were eligible, but none appear in the included studies. Clarify whether any were retrieved and screened, or whether all excluded.

While the manuscript suggests schistosomiasis screening in gynecological exams, this recommendation may be premature given the limited and heterogeneous evidence. Authors should reframe as a potential avenue for research/pilot programs, not a definitive recommendation.

My suggestion is that the author should add a table summarizing key limitations and potential biases of each included study.

Reviewer #5: The objective is clearly stated.

**********

**Results**

-Does the analysis presented match the analysis plan?

-Are the results clearly and completely presented?

-Are the figures (Tables, Images) of sufficient quality for clarity?

Reviewer #1: All the above do not holds true, as the manuscript is a review

Reviewer #2: - The results are clearly and completely presented.

- Figure 2 and Table 1 clear and useful.

- Line 229 : are there two studies that evaluated patients for "just hookworm" ? (It seems there are 6 schists / 1 "just hookworm" / 1 helminth infections.

- In table 1, association should not be marked as positive if the result is not statistically significant (consider "not significant, but positive trend").

Reviewer #3: •Please specify whether the ORs in the text and Table 1 are crude or adjusted.

•Line 249: The reported CI seems to have a typo. The OR suggests an association between HSIL and schistosomiasis, but it is not statistically significant. Please double-check.

•Line 255–256: Both Petry et al. studies showed positive associations, but they were not statistically significant. Clarify.

•Line 258–260: Reiterate that results were not statistically significant.

Reviewer #4: The Abstract section: The Results section says “all eight studies showed a positive relationship,” but one study had borderline/non-significant estimates. Adjust wording to avoid overgeneralization.

The Results emphasize “consistent positive associations,” but many effect estimates have very wide CIs crossing unity (e.g. OR 7.1 [0.5–92.1]; OR 2.83 [0.27–29.9]). Overstatement of consistency should be tempered.

Reviewer #5: The analysis match the analysis plan.

**********

**Conclusions**

-Are the conclusions supported by the data presented?

-Are the limitations of analysis clearly described?

-Do the authors discuss how these data can be helpful to advance our understanding of the topic under study?

-Is public health relevance addressed?

Reviewer #1: Yes

Reviewer #2: - The conclusions are clear and interesting.

- Limitations and gaps in knowledge are clearly discussed.

- The study presents relevant results for public health.

Reviewer #3: •Line 266: Out of nine ORs presented, five were not statistically significant. Therefore, it is not appropriate to conclude consistent positive associations; at best, there is a suggestive trend.

•Line 269: The conclusion should be stated more carefully. Without quality/risk of bias assessment, knowledge of whether ORs are crude/adjusted, and other variables (e.g., HPV vaccination status), the data cannot support a strong hypothesis.

•Line 275–276: This is a more comprehensive conclusion. Consider making this the main message.

•Line 276–277: Did the included studies report sample size calculations or inclusion of the entire population? Without this information, attributing results to small sample sizes is speculative. Other factors (biases, diagnostic precision) may also explain findings.

•Lines 316–319: Strong recommendations cannot be made without systematic reviews and meta-analyses. Current evidence is insufficient to recommend schistosomiasis screening during gynecological/obstetric check-ups.

•The authors should explicitly address whether there is evidence that treating schistosomiasis improves HPV-related lesion outcomes.

Reviewer #4: (No Response)

Reviewer #5: The conclusions are supported by the data presented.

**********

**Editorial and Data Presentation Modifications?**

Use this section for editorial suggestions as well as relatively minor modifications of existing data that would enhance clarity. If the only modifications needed are minor and/or editorial, you may wish to recommend “Minor Revision” or “Accept”.

Reviewer #1: None

Reviewer #2: (No Response)

Reviewer #3: (No Response)

Reviewer #4: The manuscript addresses an underexplored but highly relevant area. With improved balance in interpretation, clearer acknowledgment of limitations, and more cautious recommendations, it will make a strong contribution.

Reviewer #5: Minor revision.

**********

**Summary and General Comments**

Use this section to provide overall comments, discuss strengths/weaknesses of the study, novelty, significance, general execution and scholarship. You may also include additional comments for the author, including concerns about dual publication, research ethics, or publication ethics. If requesting major revision, please articulate the new experiments that are needed.

Reviewer #1: Comments

Minor comments:

1)Your methods part of abstract lacks the following description:

-Selection criteria for the articles

-The search ‘terms’ for selecting the articles

-How many articles were found and how many of them were selected based on the criteria?

2)A paragraph should consist of at least three sentences and utmost eight sentences. Therefore, divide your first long paragraph of “Introduction” into two or three paragraphs.

Sections

Abstract

Page-2

Background

L24: delete the word “Because” and replace it by “Since,”

L51:

Introduction

From page 4

L70 – L75: The two sentences require reference.

L78 – L81: Similarly, requires reference

L87 – L89: Also requires reference

Methods

From page 7 to page 9

L116: Delete the word “manuscripts” and replace it by “articles”

L161: Write the first letter of “Results” in small letter as “Synthesis of results”

Results

From page 10 to page 14

L177: Add the word “evaluation” after full text

L217: Write the first letters of words of the subheading “Characteristics of Sources of Evidence” in small letter except the first word as “Characteristics of Ssources of Eevidence”

L264: Please put the Table title of Table-1 above the Table in the line number 263

Discussion

From page 15 to page 16

L296: Delete the word “was” and replace it by “were”

L298: Add the words “the indexers” in the word between ‘in’ and ‘the’

You are left with the following headings after conclusion:

-Acknowledgements

-Abbreviation

-Authors’ contributions

Comments on references

1)Since, your 12th reference is published in anon-periodically publishing journal, you need to include the article URL at the end. The same comment holds true for your 20th, 25th, 26th, references.

2)From your 22nd reference, delete the 7th author and replace it by et al.

Reviewer #2: This review is well written and tackles an interesting issue that is not well known, with few data published for the moment. The findings are important and helpful to better understand the epidemiology and strengthen public health programs.

Reviewer #3: The evidence presented suggests a possible association between helminth prevalence and HPV/HPV-related lesions, but:

•Many associations are not statistically significant.

•The included studies are from LMICs where multiple confounders exist.

•The review lacks important methodological rigor (protocol registration, comprehensive database search, risk of bias assessment). Although critical appraisal is optional according to the Joanna Briggs Institute Manual for Evidence Synthesis and the PRISMA-ScR (Preferred Reporting Items for Systematic reviews and Meta-Analyses extension for Scoping Reviews) if you provide inferential results, these must be supported by rigorous studies that tried to minimize biases.

The discussion should more directly highlight research gaps, limitations, and the need for well-designed epidemiological studies before making recommendations.

Line 50: The statement should be more cautious. At present, there is no robust evidence that screening and treating schistosomiasis improves the clinical outcomes of women with precancerous or cancerous cervical lesions.

The recommendation should emphasize that any public health policy must be based on high-quality epidemiological evidence, which is currently lacking. For instance, greater efforts should be made in terms of HPV vaccine access and availability in populations where intestinal parasites are highly prevalent.

Figure 1: Comparing 2016 DALYs vs. 2020 incidence is not appropriate. Recommend comparing similar years and similar variables (e.g., prevalence of schistosomiasis vs. cervical cancer in the same year). IHME GBD visualization tool could be used (https://vizhub.healthdata.org/gbd-compare/). However, by using this tool, the geographical overlapping that the authors mention is not that clear.

Minor Comments

•Line 61–62: The sentence is unclear. Do the authors mean that hrHPV typically becomes undetectable within 6–18 months after acquisition? Please rephrase.

•Line 116: Revise to “manuscripts written in English or Spanish” (omit “language”).

•Line 179–180: Clarify what is meant by “eight contained inadequate information.” Inadequate for what purpose?

•Line 218–219: Swanepoel et al. seems to be a cross-sectional study. “Retrospective” is not a study design but rather a data collection direction.

•Line 225–229: The sentence is confusing. LEEP and biopsies are used to detect/treat cervical lesions, not to diagnose schistosomiasis. Please reframe.

•Line 279–282: A reference is needed to support this sentence.

•Line 367: Reference 13 appears incomplete.

Reviewer #4: This is an important and timely review addressing the potential interaction between helminth infections and HPV persistence/cervical cancer in LMICs. The manuscript is generally well structured, clear, and adheres to PRISMA-ScR guidelines. However, there are several issues in framing, methodological reporting, and interpretation that require revision to improve clarity, rigor, and balance.

Reviewer #5: I would like to thank the authors for the valuable article. I have few suggestions that I think will help the authors to further enrich the manuscript.

1- The results are clearly presented with a table summarizing the studies discussed. However, the analysis can be further enriched by the inclusion of information regarding other factors such as age of the participants, that may influence susceptibility to chronic HPV infection and cervical cancer, in these studies.

2-The conclusions are well-supported. Nevertheless, more attention should be paid on the possible immunologic mechanisms (with support of a figure) behind the association between helminths and HPV or cervical cancer. The possible effect of Th2 immune response make the reader think whether other Th2-related conditions (such as allergy) are also associated with altered susceptibility to particular infections and/or cancer. I also wonder if it is possible to compare compare persistent HPV infection and/or cervical cancer rates between regions with high and low helminth prevalence, by using WHO data, to further support the conclusions drawn.

**********

PLOS authors have the option to publish the peer review history of their article (what does this mean?). If published, this will include your full peer review and any attached files.

If you choose “no”, your identity will remain anonymous but your review may still be made public.

**Do you want your identity to be public for this peer review?**  For information about this choice, including consent withdrawal, please see our Privacy Policy.

Reviewer #1: No

Reviewer #2: No

Reviewer #3: No

Reviewer #4: **Yes:** Manfred Dakorah Asiedu

Reviewer #5: No

[NOTE: If reviewer comments were submitted as an attachment file, they will be attached to this email and accessible via the submission site. Please log into your account, locate the manuscript record, and check for the action link "View Attachments". If this link does not appear, there are no attachment files.]

**Figure resubmission:**

**Reproducibility:**

To enhance the reproducibility of your results, we recommend that authors of applicable studies deposit laboratory protocols in protocols.io, where a protocol can be assigned its own identifier (DOI) such that it can be cited independently in the future. Additionally, PLOS ONE offers an option to publish peer-reviewed clinical study protocols. Read more information on sharing protocols at https://plos.org/protocols?utm_medium=editorial-email&utm_source=authorletters&utm_campaign=protocols

# Author comment: Author response to Decision Letter 1

**DOI:** 10.1371/journal.pntd.0013308.r002

10 Mar 2026

Attachment Submitted filename: Response to Reviewers.docx

# Peer review: Decision Letter 1

**DOI:** 10.1371/journal.pntd.0013308.r003

**Authors:** Aysegul Taylan Ozkan, Krystyna Cwiklinski

22 May 2026

Response to Reviewers Revised Manuscript with Track Changes Manuscript

Shaden Kamhawi

co-Editor-in-Chief

PLOS Neglected Tropical Diseases

orcid.org/0000-0003-4304-636XX

Paul Brindley

co-Editor-in-Chief

PLOS Neglected Tropical Diseases

orcid.org/0000-0003-1765-0002

**Reviewers' comments:**

**Key Review Criteria Required for Acceptance?**

As you describe the new analyses required for acceptance, please consider the following:

**Methods**

-Are the objectives of the study clearly articulated with a clear testable hypothesis stated?

-Is the study design appropriate to address the stated objectives?

-Is the population clearly described and appropriate for the hypothesis being tested?

-Is the sample size sufficient to ensure adequate power to address the hypothesis being tested?

-Were correct statistical analysis used to support conclusions?

-Are there concerns about ethical or regulatory requirements being met?

Reviewer #1: Yes

Reviewer #3: The methods are clear and replicable.

Reviewer #5: (No Response)

**********

**Results**

-Does the analysis presented match the analysis plan?

-Are the results clearly and completely presented?

-Are the figures (Tables, Images) of sufficient quality for clarity?

Reviewer #1: Yes

Reviewer #3: The results section require minor corrections (see general comments).

Reviewer #5: (No Response)

**********

**Conclusions**

-Are the conclusions supported by the data presented?

-Are the limitations of analysis clearly described?

-Do the authors discuss how these data can be helpful to advance our understanding of the topic under study?

-Is public health relevance addressed?

Reviewer #1: Yes

Reviewer #3: The conclusions should be revised (see general comments).

Reviewer #5: (No Response)

**********

**Editorial and Data Presentation Modifications?**

Use this section for editorial suggestions as well as relatively minor modifications of existing data that would enhance clarity. If the only modifications needed are minor and/or editorial, you may wish to recommend “Minor Revision” or “Accept”.

Reviewer #1: None

Reviewer #3: See general comments

Reviewer #5: (No Response)

**********

**Summary and General Comments**

Use this section to provide overall comments, discuss strengths/weaknesses of the study, novelty, significance, general execution and scholarship. You may also include additional comments for the author, including concerns about dual publication, research ethics, or publication ethics. If requesting major revision, please articulate the new experiments that are needed.

Reviewer #1: Comments

Minor comments

- The figure caption of Figure-1 is almost a paragraph. Make it short and precise.

- Re-arrange your abbreviations on page-19 in alphabetical order

Specific comments

Page-5

L95: insert the word “enrich” between ‘further’ and ‘our’

Results

Page-12

L248 – L249 – write the full form of CIN and HSIL with the abbreviated for the first time and then you can use the abbreviated form only. The same comment holds true for LEEP in L251

Page-13

L273: delete the letter “a” found between the words ‘but’ and ‘suggests’

Discussion

Page-18

L343: delete “HrHPV” and replace it by “hrHPV”

Comments on references

1. The number of authors in your 10th reference is 7. Therefore, delete the 7th and replace it by et al. The same comment holds true for your 13th, 16th, 25th, 26th, references.

2. Your 11th reference lacks page number; include the page number. The same comment holds true for your 19th reference. The same comment holds true for your 20th, references.

3. Since your 24th reference is published in a non-periodically publishing journal, delete the initial page number “691” and replace it by the article URL.

Reviewer #3: Dear authors,

I have reviewed the final version of the manuscript titled: Understanding the association between immune modulating helminths and HPV or cervical cancer: a scoping review. The authors have addressed most comments and recommendations. However, some minor corrections should be made before the manuscript is published in PLOS Neglected Tropical Diseases.

Prefer “immunomodulatory” or “immune-modulating” instead of “immune modulating” in the title and running text.

Line 166: Change to synthesized.

Line 259: The authors stated: “...though some lacked statistical significance”. However, most (5 out of eight studies) lacked statistical significance.

Lines 265-268: The authors cited Swanepoel et al study. It is important to mention that the result from this study was not statistically significant.

Line 271: Fix the CI. The lower confidence bound is incorrect (OR: 2.76; 95% CI: 2.76–8.46)}

Line 276-278: The confidence interval contains the null value (1.0). Therefore, change the verb (demonstrate) to suggest. Additionally, table 1 states that the effect measurement used was a prevalence ratio while the text says OR. Clarify it.

Line 293: The authors stated: “Although not all effect measurements were statistically significant…”. To be more precise indicate that most effect measures (again, 5 out of eight studies) were not statistically significant.

Line 303-305: They might not be statistically significant exclusively to a small sample size. Did all the included studies calculated a sample size? Were the sample sizes inadequate to test the current study hypothesis? Additionally, take into account that the results might be bias due to the observational nature of the included studies and that those studies could have other main outcomes not related to this scoping review.

Line 343: Use “hrHPV” consistently (avoid “HrHPV”).

Lines 353-356: The authors recommend frequent (every three months) de-worming of reproductive age individuals. The proposed frequency is not consistent with core WHO preventive chemotherapy recommendations, which commonly reference annual or biannual schedules depending on baseline prevalence and target groups (including women of reproductive age) https://www.who.int/publications/i/item/9789241550116

Line 166: Change to deworming.

Prefer “antiviral” over “anti-viral.

Add missing abbreviations in the abbreviations list:

• PLWH

• NTDs

• WASH

Table 1: Not all association can be interpreted as positive using an asterisk. Either some are positive and the rest are null, or all are positive and only those which confidence intervals include the null value should include the asterisk.

Consider adding the extracted 2×2 tables from studies that did not report effect measurements (these should be placed in Supplemental Information).

Reviewer #5: (No Response)

**********

PLOS authors have the option to publish the peer review history of their article (what does this mean?). If published, this will include your full peer review and any attached files.

If you choose “no”, your identity will remain anonymous but your review may still be made public.

**Do you want your identity to be public for this peer review?**  For information about this choice, including consent withdrawal, please see our Privacy Policy.

Reviewer #1: **Yes:** Dr. Ephrem Tefera Solomon

Reviewer #3: No

Reviewer #5: No

**Figure resubmission:**

**Reproducibility:** -->To enhance the reproducibility of your results, we recommend that authors of applicable studies deposit laboratory protocols in protocols.io, where a protocol can be assigned its own identifier (DOI) such that it can be cited independently in the future. Additionally, PLOS ONE offers an option to publish peer-reviewed clinical study protocols. Read more information on sharing protocols at https://plos.org/protocols?utm_medium=editorial-email&utm_source=authorletters&utm_campaign=protocols

Attachment Submitted filename: Reviewers comments.docx

# Author comment: Author response to Decision Letter 2

**DOI:** 10.1371/journal.pntd.0013308.r004

30 Jun 2026

Attachment Submitted filename: Response_to_reviewers_auresp_2.docx

# Peer review: Decision Letter 2

**DOI:** 10.1371/journal.pntd.0013308.r005

**Authors:** Aysegul Taylan Ozkan, Krystyna Cwiklinski

13 Jul 2026

Response to Reviewers Revised Manuscript with Track Changes Manuscript

Shaden Kamhawi

co-Editor-in-Chief

PLOS Neglected Tropical Diseases

orcid.org/0000-0003-4304-636XX

Paul Brindley

co-Editor-in-Chief

PLOS Neglected Tropical Diseases

orcid.org/0000-0003-1765-0002

**Additional Editor Comments (if provided):** **Journal Requirements:**

- A numbered table of all studies identified in the literature search, including those that were excluded from the analyses.

- For every excluded study, the table should list the reason(s) for exclusion.

- If any of the included studies are unpublished, include a link (URL) to the primary source or detailed information about how the content can be accessed.

- A table of all data extracted from the primary research sources for the systematic review and/or meta-analysis. The table must include the following information for each study:

- Name of data extractors and date of data extraction

- Confirmation that the study was eligible to be included in the review.

- All data extracted from each study for the reported systematic review and/or meta-analysis that would be needed to replicate your analyses.

- If data or supporting information were obtained from another source (e.g. correspondence with the author of the original research article), please provide the source of data and dates on which the data/information were obtained by your research group.

- If applicable for your analysis, a table showing the completed risk of bias and quality/certainty assessments for each study or outcome.  Please ensure this is provided for each domain or parameter assessed. For example, if you used the Cochrane risk-of-bias tool for randomized trials, provide answers to each of the signalling questions for each study. If you used GRADE to assess certainty of evidence, provide judgements about each of the quality of evidence factor. This should be provided for each outcome.

- An explanation of how missing data were handled.

This information can be included in the main text, supplementary information, or relevant data repository. Please note that providing these underlying data is a requirement for publication in this journal, and if these data are not provided your manuscript might be rejected.

**Reviewers' comments:**

**Key Review Criteria Required for Acceptance?**

As you describe the new analyses required for acceptance, please consider the following:

**Methods**

-Are the objectives of the study clearly articulated with a clear testable hypothesis stated?

-Is the study design appropriate to address the stated objectives?

-Is the population clearly described and appropriate for the hypothesis being tested?

-Is the sample size sufficient to ensure adequate power to address the hypothesis being tested?

-Were correct statistical analysis used to support conclusions?

-Are there concerns about ethical or regulatory requirements being met?

Reviewer #3: Yes

**********

**Results**

-Does the analysis presented match the analysis plan?

-Are the results clearly and completely presented?

-Are the figures (Tables, Images) of sufficient quality for clarity?

Reviewer #3: Yes

**********

**Conclusions**

-Are the conclusions supported by the data presented?

-Are the limitations of analysis clearly described?

-Do the authors discuss how these data can be helpful to advance our understanding of the topic under study?

-Is public health relevance addressed?

Reviewer #3: Yes

**********

**Editorial and Data Presentation Modifications?**

Use this section for editorial suggestions as well as relatively minor modifications of existing data that would enhance clarity. If the only modifications needed are minor and/or editorial, you may wish to recommend “Minor Revision” or “Accept”.

Reviewer #3: See general comments

**********

**Summary and General Comments**

Use this section to provide overall comments, discuss strengths/weaknesses of the study, novelty, significance, general execution and scholarship. You may also include additional comments for the author, including concerns about dual publication, research ethics, or publication ethics. If requesting major revision, please articulate the new experiments that are needed.

Reviewer #3: I would like to thank the authors for carefully addressing my previous comments and for further improving their manuscript based on my recommendations. The manuscript is now suitable for publication in PLOS Neglected Tropical Diseases. Before publication, however, I recommend that the authors address the following minor comments:

The authors agreed to use “immune-modulating” instead of “immune modulating” in both the title and the short title. However, these changes are not reflected in the current version of the manuscript.

Line 105: The manuscript states that the scoping review was conducted in collaboration with an NIH librarian (AL), whereas Line 126 refers to the librarian as AAL. Please ensure consistency throughout the manuscript.

The following abbreviations are still missing from the abbreviations list: PLWH, NTDs, and WASH.

The authors previously agreed that using an asterisk in Table 1 could be misleading. However, the current version places an asterisk next to all results rather than only those positive associations whose confidence intervals include the null value and therefore did not reach statistical significance. Please review the use of the asterisk and revise the table accordingly.

**********

PLOS authors have the option to publish the peer review history of their article (what does this mean?). If published, this will include your full peer review and any attached files.

If you choose “no”, your identity will remain anonymous but your review may still be made public.

**Do you want your identity to be public for this peer review?**  For information about this choice, including consent withdrawal, please see our Privacy Policy.

Reviewer #3: No

**Figure resubmission:**

**Reproducibility:**

To enhance the reproducibility of your results, we recommend that authors of applicable studies deposit laboratory protocols in protocols.io, where a protocol can be assigned its own identifier (DOI) such that it can be cited independently in the future. Additionally, PLOS ONE offers an option to publish peer-reviewed clinical study protocols. Read more information on sharing protocols at https://plos.org/protocols?utm_medium=editorial-email&utm_source=authorletters&utm_campaign=protocols

# Author comment: Author response to Decision Letter 3

**DOI:** 10.1371/journal.pntd.0013308.r006

3 Aug 2026

Attachment Submitted filename: Response to reviewers 7.22.26.docx

# Editor report: Decision Letter 3

**DOI:** 10.1371/journal.pntd.0013308.r007

**Authors:** Aysegul Taylan Ozkan, Krystyna Cwiklinski

10 Aug 2026

Dear Correa-Mendez,

We are pleased to inform you that your manuscript 'Understanding the association between immune-modulating helminths and HPV or cervical cancer: a scoping review' has been provisionally accepted for publication in PLOS Neglected Tropical Diseases.

Before your manuscript can be formally accepted you will need to complete some formatting changes, which you will receive in a follow up email. A member of our team will be in touch with a set of requests.

Please note that your manuscript will not be scheduled for publication until you have made the required changes, so a swift response is appreciated.

IMPORTANT: The editorial review process is now complete. PLOS will only permit corrections to spelling, formatting or significant scientific errors from this point onwards. Requests for major changes, or any which affect the scientific understanding of your work, will cause delays to the publication date of your manuscript.

Should you, your institution's press office or the journal office choose to press release your paper, you will automatically be opted out of early publication. We ask that you notify us now if you or your institution is planning to press release the article. All press must be co-ordinated with PLOS.

Thank you again for supporting Open Access publishing; we are looking forward to publishing your work in PLOS Neglected Tropical Diseases.

Best regards,

Aysegul Taylan Ozkan, M.D., Ph.D.,

Academic Editor

PLOS Neglected Tropical Diseases

Krystyna Cwiklinski

Section Editor

PLOS Neglected Tropical Diseases

Shaden Kamhawi

co-Editor-in-Chief

PLOS Neglected Tropical Diseases

orcid.org/0000-0003-4304-636XX

Paul Brindley

co-Editor-in-Chief

PLOS Neglected Tropical Diseases

orcid.org/0000-0003-1765-0002

***********************************************************

p.p1 {margin: 0.0px 0.0px 0.0px 0.0px; line-height: 16.0px; font: 14.0px Arial; color: #323333; -webkit-text-stroke: #323333}span.s1 {font-kerning: none

# Editor report: Acceptance letter

**DOI:** 10.1371/journal.pntd.0013308.r008

**Authors:** Aysegul Taylan Ozkan, Krystyna Cwiklinski

Dear Correa-Mendez,

We are delighted to inform you that your manuscript, "Understanding the association between immune-modulating helminths and HPV or cervical cancer: a scoping review," has been formally accepted for publication in PLOS Neglected Tropical Diseases.

We have now passed your article onto the PLOS Production Department who will complete the rest of the publication process. All authors will receive a confirmation email upon publication.

The corresponding author will soon be receiving a typeset proof for review, to ensure errors have not been introduced during production. Please review the PDF proof of your manuscript carefully, as this is the last chance to correct any scientific or type-setting errors. Please note that major changes, or those which affect the scientific understanding of the work, will likely cause delays to the publication date of your manuscript. Note: Proofs for Front Matter articles (Editorial, Viewpoint, Symposium, Review, etc...) are generated on a different schedule and may not be made available as quickly.

Soon after your final files are uploaded, the early version of your manuscript will be published online unless you opted out of this process. The date of the early version will be your article's publication date. The final article will be published to the same URL, and all versions of the paper will be accessible to readers.

For Research Articles, you will receive an invoice from PLOS for your publication fee after your manuscript has reached the completed accept phase. If you receive an email requesting payment before acceptance or for any other service, this may be a phishing scheme. Learn how to identify phishing emails and protect your accounts at https://explore.plos.org/phishing.

Thank you again for supporting open-access publishing; we are looking forward to publishing your work in PLOS Neglected Tropical Diseases.

Best regards,

Shaden Kamhawi

co-Editor-in-Chief

PLOS Neglected Tropical Diseases

Paul Brindley

co-Editor-in-Chief

PLOS Neglected Tropical Diseases
